# The influence of lipid content and pretreatment methods on protein conformation in fish (capelin, *Mallotus villosus*) during smoking and drying

**DOI:** 10.1002/fsn3.980

**Published:** 2019-02-27

**Authors:** Cyprian Ogombe Odoli, Peter Oduor‐Odote, Sigurjon Arason

**Affiliations:** ^1^ Kenya Marine & Fisheries Research Institute, Baringo Station Kampi ya Samaki Kenya; ^2^ Kenya Marine & Fisheries Research Institute, Mombasa Centre Mombasa Kenya; ^3^ Department of Food Science University of Iceland Reykjavik Iceland; ^4^ Matis ohf./Icelandic Food and Biotech Research & Development Reykjavik Iceland

**Keywords:** drying, fish, protein conformation, smoking

## Abstract

The influence of lipid content and pretreatment methods (blanching and brining) on proteins conformation in fish (Capelin, *Mallotus *v*illosus*) during drying and smoking were assessed. Soluble protein fractions were examined through changes in salt‐soluble proteins, sulfhydryl groups, and disulfide bonds contents. The salt level and moisture content were also evaluated. Conformational changes in muscle protein were significant (*p* < 0.05) after blanching and during initial drying when moisture content and dryness rates were high. Salt‐soluble proteins and total sulfhydryl groups contents reduced, whereas available sulfhydryl group and disulfide bonds contents intensified. The conformational changes were explained by muscle protein denaturation and aggregation ascribed to high temperature and dehydration during processing. The study reports fish protein denaturation to have reduced protein solubility as well as the nutritional value (loss of thermolabile compounds) and yield after smoking. More so, protein aggregation that was significantly (*p* < 0.05) higher in dried blanched fish could have reduced the dried fish sensory quality. Blanching pretreatment is thus not suitable for commercial capelin drying. Lipids were found to have protective effect against fish protein conformation.

## 1 INTRODUCTION

Fish drying and smoking are preservation methods commonly used in most developing nations (Akintola, Brown, Bakare, Osowo, & Omolola, 2013; Bellagha, Sahli, Farhat, Kechaou, & Glenza, 2007; Darvishi, Azadbakht, Rezaeiasl, & Farhang, 2013). In some countries, fish is brined or partially boiled (blanching) in brine before drying to inactivate enzymes and inhibit growth of bacteria. While smoking is commonly conducted either by hot smoking or cold smoking method depending on fish species and type of products desired, smoking changes the appearance, taste, and odor of the product, in addition to increasing the shelf life (Cyprian et al., 2015).

Earlier studies report fish muscle protein to be less stable than ones originating from mammals (Baylan et al., 2015; Poulter, Ledward, Godber, Hall, & Rowlands, 1985). Fish drying or smoking process is therefore believed to have a consequence on muscle protein solubility and aggregation, thereby affecting the protein structure in the intracellular and extracellular matrix (Nguyen, Thorarinsdottir, Gudmundsdottir, Thorkelsson, & Arason, 2011). The extent of protein denaturation and aggregation could depend on smoking (i.e., hot smoking or cold smoking method) and drying methods as well as pretreatment (i.e., brining or blanching) before processing (drying and smoking). The chemical composition of fish, particularly lipid content, is thought to contribute greatly on progression of protein denaturation/aggregation. Rustad and Nesse (1983) described fish muscle protein denaturing temperature to be dependent mainly on the species. The warm water (tropical) species comprise proteins that are thermally stable than those of temperate fish species (Meneshi, Harwood, & Grant, 1976; Poulter et al., 1985). Murueta Toro and Carreño (2007) described proteins denaturation temperature of nearly 90% of tropical fish to be between 60 and 65°C.

Conformational changes in proteins arising due to denaturation and aggregation are associated mainly with changes in secondary, tertiary, and quaternary structure (Nguyen et al., 2011), but also the disulfide bonds formation (Raman & Mathew, 2014). Changes occur mainly in the reactive groups, when hydrophilic surface is reduced, and hydrophobic and sulfhydryl groups buried or blocked in native proteins are exposed (Baylan et al., 2015; Nguyen et al., 2011). During heating (i.e., drying and smoking) secondary, tertiary, and quaternary structures of protein may be lost owing to split in the hydrogen bonds resulting from unfolding of the native structure (Baylan et al., 2015; Raman & Mathew, 2014). The protein functional properties are largely defined by the conformation changes occurring (Ghelichpour & Shabanpour, 2011). This is because conformational changes in protein may destroy portion of their physical and chemical properties (Rustad & Nesse, 1983; Skipnes, Van der Plancken, Van Loey, & Hendrickx, 2008). Thus reducing the nutritional value, for example, loss of thermolabile compounds (amino acids) and capacity to hold water, affecting the product's textural properties (Raman & Mathew, 2014).

The consequence of lipid content and fish blanching prior to drying on muscle proteins during drying and smoking is ill understood. Furthermore, dried and smoked small fish traded as human food in certain developing nations are contrarily to capelin, caught in tropical/warm water. Blanching, drying, and smoking could have a greater effect on fish muscle proteins of temperate than tropical species. This could affect the eating and sensory quality, precisely texture, nutritive worth, and yield which are affected by protein conformation. The aim of the present study was to assess the influence of lipid content and pretreatment methods (blanching in brine and brining) on the protein conformation changes in capelin during drying and smoking. Protein changes in fish muscle were monitored through changes in the salt‐soluble proteins (SSP), sulfhydryl (SH) groups’, and disulfide content (S–S). Understanding the effect of blanching and brining as well as lipid content on protein conformation would be valuable in establishing suitable pretreatment before processing and desired lipid level in maintaining nutritional value while upholding yield and sensory properties for smoked and dried small pelagic fish species.

## 2 MATERIALS AND METHODS

### 2.1 Raw material, processing, and sampling

Two groups of capelin (*Mallotus *v*illosus*) caught on February 13, 2015 and March 7, 2015 were kept chilled in seawater for 2 days prior to grading and freezing in 25 kg blocks. Individual fish/piece weighed 32 ± 4 g. The fish were obtained from a fishing company (HB Grandi) in Reykjavik—Iceland and stored frozen at −25°C for 3 months to the study time. Prior to the experimental set up, fish was thawed (overnight) at 18–20°C in open air. After thawing, each group was subdivided into four equivalent portions. Half the portions were either blanched in brine or brined (pretreatment) prior to drying. The remainder (two portions) were brined before either cold or hot smoking. Brining was carried out by immersing the portions separately in 5% sodium chloride (NaCl salt) solution for 2 hr at 2°C to attain around 2% salt level in the muscle. Afterward, samples were placed in a single layer on inclined trays (meshed) to drip dry. Portions for blanching pretreatment were separately placed in metallic pans with perforations and immersed in boiling water (5% NaCl salt) for 2 min, removed, and spread on meshed trays to drip dry and cool before subsequent processing. The trays with fish were stacked on drying and smoking racks.

Fish was dried in a tunnel drier with controlled temperatures (22 ± 2°C), air speed (3 ± 0.5 m/s), and relative humidity (40 ± 5%) at Vestfirska Hardfisksalan fish drying company (Reykjavik, Iceland). Eighteen individual fish (6 pooled in a bag) from each group were sampled at every point (0, 17, 24, 48, 72, 112, 160, & 184 hr during drying). Hot and cold smoking was conducted in a conventional kiln with automatic controls for humidity, temperature, and smoke density. Hot smoking was conducted in three stages of 2 hr initial smoking at 30°C, then 2 hr smoking at 40°C and 30 min smoking at 75°C (Cyprian et al., 2016). Eighteen individual fish (6 pooled in a bag) from each group were collected at air refreshing time subsequent to the second cycle during both first and second stages, and at the end of the third stage in hot smoking. Whereas cold smoking was carried out for 4 hr at 24°C, and samples were collected at refreshing time following second, fourth, and sixth cycles, and at the end of smoking (Cyprian et al., 2016). Collected samples were packaged in polyethylene bags and stored at −80°C until studied.

### 2.2 Chemicals

The chemicals used in the study were purchased from Sigma‐Aldrich (St. Louis, MO, USA) and Fluka (Buchs, Switzerland) and were analytical grade.

### 2.3 Moisture content, salt, and lipid content

Moisture content of raw and processed samples was determined as the weight reduction of minced samples after drying at 103 ± 1°C for 4 hr (ISO, 1993), and results expressed as gram water/100 g sample mince. The samples salt content/level (NaCl) was established as described in AOAC (2000) and outcomes expressed as gram salt/100 g sample mince. The lipids were extracted from 25 g of sample mince (80 ± 1% water) (*n* = 3) with methanol/chloroform/0.88% KCl_(aq)_ (at 1/1/0.5, v/v/v) (Bligh & Dyer, 1959). Lipid content was determined gravimetrically, and the results were conveyed as g lipid per 100 g sample mince.

### 2.4 Salt‐soluble proteins extraction

The salt‐soluble proteins (SSP) were determined by mincing samples in a Braun Mixer (Type 4262, Germany) for 2–3 min. The mince (10 g) was added to 190 ml of salt solutions (1 M NaCl and 0.02 M Na_2_CO_3_, pH 7.0) as described by Kelleher and Hultin (1991) with modifications in buffer. The resultants were homogenized at 6,000 rpm for 1 min using an Ultra‐turrax homogenizer (Ika Labortechnik, T25 basic, Staufen, Germany) and homogenates kept chilled on ice for 1 hr before centrifugation in an Avanti Centrifuge J‐20 XPI (Beckmann Coulter, Fullerton, CA, USA) for 15 min (at 0–5°C) at 10,000 rpm. The amount of protein solubilized in the supernatant was quantified based on the Bradford method (Bradford, 1976), adapted to microassays using bovine serum albumin as a standard. The diluted supernatant and the Bradford reactive were placed in a 96‐well microplate and absorbance measured in a Sunrise Microplate Reader (Tecan GmbH, A‐5082 Grödig, Austria) at 595 nm.

### 2.5 Disulfide bond content

The disulfide bond content in protein was assayed (Thannhauser, Konishi, & Scheraga, 1984) using 2‐nitro‐5‐thiosulfobenzoate (NTSB). The supernatants from NaCl protein extractions were diluted to protein concentration of 0.1 mg/ml. Three ml of freshly prepared NTSB assay solution, pH 9.5 (adjusted with 0.1 M HCl or 0.1 M NaOH) were added to 0.25 ml protein solution (0.1 mg/ml) in a 4.5 ml cuvette. The blend was incubated at room temperatures for 25 min in a dark place and the absorbance read at 412 nm in spectrophotometer (UV—1800, Shimadzu, Kyoto, Japan) at 412 nm. The disulfide bond content was determined using a molar extinction coefficient of 13,900 M^−1^/cm^−1^ and expressed as µmol SS/g protein.

### 2.6 Sulfhydryl group content

The sulfhydryl (SH) groups content were determined using 5,5′‐dithiobis‐(2‐nitrobenzoic acid) (DTNB), according to the method of Beveridge, Toma, and Nakai (1974). The supernatant from NaCl protein extractions were diluted to the concentration of 0.1 mg/ml that were used in analyzing total SH and available SH groups. For the total SH content, 0.5 ml of the protein solution were added to 2.5 ml of Tris–SDS buffer pH 8.0 (0.1 M Tris, 3 mM EDTA, 3% sodium dodecyl sulfate (SDS), 0.1 M glycine and 8 M urea). To the mixture, 0.1 ml of Ellman's reagent (2 mM 5,50‐dithiobis‐(2‐nitrobenzoic acid) were added and mixed before incubating in darkness at room temperature for 30 min. After incubation, the solutions absorbance was measured at 412 nm (UV—1800 spectrophotometer, Shimadzu, Kyoto, Japan). The total SH groups’ content was calculated using a molar extinction coefficient of 13,900 M^−1^ cm^−1^. The available SH content was determined as with total SH content except that 0.5 ml of protein solution was added to 2.5 ml of Tris buffer pH 8.0 (0.1 M Tris, 3 mM EDTA, 0.1 M glycine and 8 M urea). The SH content was expressed as µmol SH/g protein.

### 2.7 Data analysis

Data analyses were done in Microsoft Excel 2010 (Microsoft Inc. Redmond, WA, USA). The results are conveyed as means (± standard deviation). One way analysis of variance (ANOVA), Duncan's post hoc test, and Pearson correlation analysis were executed on means of the variable in statistical program NCSS 2000 (NCSS, Utah, USA). In the study, *p* values of <0.05 were considered significant.

## 3 RESULTS AND DISCUSSION

### 3.1 Lipids, moisture, and salt contents

Capelin differed in lipid content depending on the time it was caught (Table 1). Fish caught in February (13th) had higher lipid content (9.1%) than ones caught at the beginning of March (7th) that recorded 7% lipid content. The groups are henceforth denoted as fatty (C1) and less fatty (C2) capelin, respectively. The moisture content was, however, inversely correlated to lipid, rising from 76.8% in mid‐February to 78.2% early March. This was expected since lipid and moisture contents in fish are known to exhibit an inverse relationship. A significant (*p* < 0.05) reduction in lipid content was recorded after blanching (Table 1). This occurrence as reported by Cyprian et al. (2015) could have been occasioned by exudation of lipids during fish blanching and drip‐drying. As noted with lipid, the moisture content was reduced significantly (*p* < 0.05) following blanching. This occurrence can be ascribed to reduced water holding capacity of myofibrillar proteins due to denaturation and hydrolysis that may have occurred while blanching. Raw capelin batches recorded salt content values of 0.72 (C1) and 0.68 (C2g) salt/100 g mince that were not significantly (*p* > 0.05) different (Table 1). After pretreatment methods (brining and blanching in brine), a significant (*p* < 0.05) increase in salt content was recorded in C2 and blanched capelin. This can be elucidated by the variances in concentration gradients between the fish muscle and the surrounding medium resulting from differences in chemical compositions (e.g., water, salt, and lipid content) of raw material. The C2 capelin had higher moisture level proposing a higher concentration gradient that resulted in higher diffusion of salt ions into C2 than C1 muscles under comparable brine level (5% NaCl). The higher salt content in C1 than C2 fish upon brining and blanching in brine could as well suggest lipids to have acted as obstacles to salt diffusion as it was slower in C1 fish.

**Table 1:** Lipid, salt, and water content of raw and processed (dried and smoked) capelin differing in lipid content (*n* = 3, 18 pieces [6 pooled together, in 3 bags]; Mean ± *SD*)

Capelin batch	Variable	Raw material	Pretreatment		Dried products		Smoked products	
			Brined	Blanched‐Brined^1^	Brined	Blanched‐Brined	Brined‐Cold	Brined‐Hot
High lipid (C1)	Lipid^2^	9.05 ± 0.2^a‐d^	8.98 ± 0.44^a‐d^	7.39 ± 0.02^a‐d^	28.13 ± 0.50^a‐d^	27.06 ± 0.73^a‐d^	—	—
	Salt	0.72 ± 0.04^a‐d^	1.95 ± 0.18^a‐d^	2.15 ± 0.02^a‐d^	6.2 ± 0.2^a‐d^	7.6 ± 0.13^a‐d^	—	—
	Water	76.8 ± 0.08^a‐d^	76.91 ± 1.05^a‐d^	74.83 ± 0.85^a‐d^	20.43 ± 1.01^a‐d^	12.03 ± 0.5^a‐d^	—	—
Low lipid (C2)	Lipid	6.99 ± 0.25^a‐d^	6.75 ± 0.12^a‐d^	6.28 ± 0.22^a‐d^	27.18 ± 0.11^a‐d^	27.41 ± 0.77^a‐d^	—	—
	Salt	0.68 ± 0.02^a‐d^	2.36 ± 0.02^a‐d^	2.41 ± 0.07^a‐d^	8.8 ± 0.1^a‐d^	9.6 ± 0.28^a‐d^	—	—
	Water	78.18 ± 1.05^a‐d^	79.13 ± 1.55^a‐d^	76.06 ± 0.95^a‐d^	19.11 ± 0.55^a‐d^	10.27 ± 0.74^a‐d^	—	—
High lipid (C1)	Lipid	9.05 ± 0.2^a‐d^	8.98 ± 0.44^a‐d^	—	—	—	15.95 ± 0.52^a‐d^	17.75 ± 60^a‐d^
	Salt	0.72 ± 0.04^a‐d^	1.95 ± 0.18^a‐d^	—	—	—	3.45 ± 0.44^a‐d^	3.81 ± 0.35^a‐d^
	Water	76.8 ± 0.08^a‐d^	76.91 ± 1.05^a‐d^	—	—	—	59.14 ± 1.52^a‐d^	54.52 ± 0.75^a‐d^
Low lipid (C2)	Lipid	6.99 ± 0.25^a‐d^	6.75 ± 0.12^a‐d^	—	—	—	13.24 ± 0.66^a‐d^	15.21 ± 1.00^a‐d^
	Salt	0.68 ± 0.02^a‐d^	2.36 ± 0.02^a‐d^	—	—	—	4.25 ± 0.25^a‐d^	4.7 ± 0.23^a‐d^
	Water	78.18 ± 1.05^a‐d^	79.13 ± 1.55^a‐d^	—	—	—	57.8 ± 1.22^a‐d^	53.67 ± 0.78^a‐d^

Notes

—, Reported earlier/later.

^a‐d^ Different letters within a row indicate significantly different values between samples (*p* < 0.05).

^1^ Pretreatment done only for drying trials.

^2^ Lipid (% lipid content), Salt (% lipid content), water (% content).

Drying experiment was conducted using both pretreated samples (brined and blanched), while only brined samples were used in smoking experiments. After processing experiments (drying and smoking), the lipid and salt levels rose, while moisture content dropped (Table 1). The increase in lipid and salt levels were higher in dried than in smoked fish, an occurrence attributed to dryness as smoked samples kept much moisture than the dried ones. Differences were also manifested between C1 and C2 samples similarly processed (either dried or smoked). As would be expected, the C2 dried faster than C1 samples, similarly the blanched dried faster than brined ones. The hot smoked samples were more dehydrated than cold smoked ones. After drying, the brined and blanched samples had comparable lipid content (*p* ˃ 0.05), but moisture and salt levels were significantly (*p* < 0.05) different. The comparable lipid content in brined and blanched samples after smoking may be elucidated by the dryness level and lipid exudation that occurred during and after blanching pretreatment. As the fish dried (became drier), the relative amount of lipid increased. Blanched fish were more dehydrated resulting in comparable lipid level with the brined samples. Capelin exhibited significant difference (*p* < 0.05) in salt content based on pretreatment. As would be expected, the higher dehydrated fish was the greater the salt level recorded.

### 3.2 Salt‐soluble proteins

**Figure 1:**
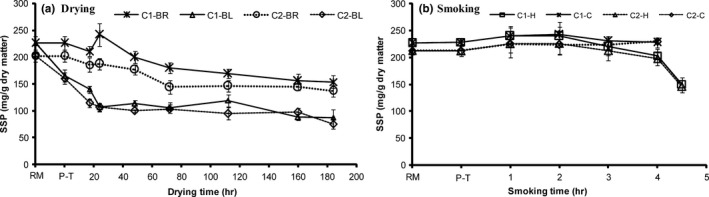
Changes in salt‐soluble proteins (SSP) in high lipid (C1) and low lipid (C2) capelin during drying and smoking (*n* = 3, 18 pieces [6 pooled together, in 3 bags]; Mean ± *SD*). Extraction of the protein fraction was performed in 1 M salt solutions. BL, blanched; BR, brined; C, cold smoking; H, hot smoking; P‐T, pretreated; RM, raw material

Lipid level, pretreatments, and processing methods (i.e., drying and smoking methods) affected salt‐soluble proteins (SSP) in samples but the manifestation varied (Figure 1a,b). A significant (*p* < 0.05) reduction in SSP was recorded in blanched fish (both C1 and C2) after brining, but it slightly increased in brined samples (both C1 and C2; Figure 1a). During drying, the SSP in all samples decreased with drying time, but was significantly (*p* < 0.05) lower in blanched when compared to brined samples. Salt‐soluble proteins values of less fatty capelin were lower than those of fatty capelin (C2‐brined vs. C1‐brined and C2‐blanched vs. C1‐blanched) throughout the process. After blanching in brine, the decrease in SSP is believed to have occurred because of heat‐induced proteins conformational changes (Finot, 1997; Ghelichpour & Shabanpour, 2011). Conformational changes in proteins resulted in losses of hydrophilic surfaces and increased hydrophobicity groups, leading to decrease SSP. The results indicate that salt uptake into the fish muscle during brining and blanching did not have any effects on the SSP, mainly due to low salt content (<2.5%) in the fish muscle. At low salt concentrations (<1 M), salt can cause the expansion of the filament lattice and depolymerization of myosin, resulting to proteins swelling due to salting—in effect (Nguyen et al., 2011). This mechanism explains why the SSP of brined samples slightly increased after brining. Comparable results were recorded prior to capelin smoking in which only brined fish was used (Figure 1b).

During drying, protein–water interactions may have been altered, exposing protein‐molecules to a less polar environment. When moisture level reduces in fish, the distance between proteins (chains) decreases (Rustad & Nesse, 1983) in that way it enhances the cross‐linkages formation between the chains (Nguyen et al., 2011). The cross‐linkages formed may cause a tighter linkage of proteins disturbing the structure and integrity (Wang, Nema, & Teagarden, 2010), resulting in intrinsic protein particles/aggregates (Fennema, 1977; Nguyen et al., 2011) reducing their solubility. The decrease in SSP can also be associated with lipid oxidation and degradation products for example malonaldehyde. The protein components (mainly peptides and acid amines) forms complex interactions through cross‐linking with oxidative degradation products resulting in intrinsic particles/aggregates (Underland, Hall, & Lingnert, 1999). Cyprian et al. (2015) reported oxidation products for instance malonaldehyde to be higher at drying onset but reduces as drying progresses. The study linked the reduction in malonaldehyde to its interactions with peptides and acid amines yielding tertiary oxidative products. The increase in salt concentration in fish muscle resulting from dehydration or induced protein conformation (i.e., aggregation) explains the decrease in SSP with drying.

During smoking, SSP were less affected by brining and were seemingly lower in C2 than C1 samples (Figure 1b). The protein solubility was observed to have been stable throughout smoking process (hot and cold smoking) with the exception of a slight increase recorded during the early smoking time (first 2 hr) and significantly lower values (*p* < 0.05) toward the end of hot smoking, recorded in both batches. A slight increase in SSP was observed during the initial smoking. The observation could be due to breakdown/hydrolysis of proteins into smaller molecular weight peptides caused by enzymatic activity and heat (Stoknes et al., 2005). Generally, the results depict trivial denaturation to have occurred in proteins during initial smoking (both cold and hot) when the temperatures were ≤30°C. The dissimilarity in SSP recorded can therefore be accounted for by the variance in lipid level between batches. Lipid appears to have a shielding outcome on protein as SSP was the highest in C1 capelin (fatty batch), supposedly due to the low heat transmission coefficient of fats. A previous study by Dyer and Dingle (1961) noted lower SSP in lean (<1% fat) than fatty (3%–10% fat) smoked fish. More so, in the present study lipids acted as a barrier to salt intake with C1 capelin obtaining lower salt level when compared to C2 capelin. Nguyen et al. (2011) found fish protein solubility and water holding capacity to be influenced by salt. However, the changes in SSP recorded during hot smoking (but not cold smoking) may principally be accredited to temperature rather than the salting‐in effect as salt was <5% in all groups.

Fish recorded reduced SSP toward the termination of hot smoking process (last 30 min) when temperature was high (at 70°C; Figure 1b). This was ascribed to the heat‐induced protein denaturation and aggregation. Murueta Toro and Carreño (2007) observed about 90% of fish muscle proteins to have been denatured between temperatures of 60 and 65°C. In the present study, fish protein denaturation initiated at smoking temperature of 40°C. However, the rate of denaturation became significantly (*p* < 0.05) higher at 75°C. This is mainly a credited to the difference in fish species used in both studies, comprising unlike protein types and thus the thermal stability. Poulter Ledward Godber Hall and Rowlands (1985) reported the proteins of warm water fish to be thermally stable than those of cold water species, since the stability of tissue proteins is known to be influenced by the habitat temperature (Meneshi et al., 1976). Capelin being a temperate (cold water/higher latitude species) explains why protein denaturation initiated at low smoking temperature (40°C). In both tested processing methods, a positive correlation was recorded between SSP and moisture content (Table 2). Stressing protein solubility decreased with declining moisture content, with a stronger correlation (*r* = 0.71) obtained in drying than during smoking (*r* = 0.61). The SSP and temperature recorded the strongest inverse relationship (*r* = −0.82) during smoking, suggesting proteins conformation that occurred while smoking was principally temperature driven.

**Table 2:** Correlation (Pearson) matrix for several parameters evaluated for dried and smoked capelin differing in lipid content

	Temp	MC	SSP	Tot‐SH	Av‐SH	Disul
A: Drying (*p* = 0.38)						
DT		**−0.91** *	**−0.53**	**−0.63**	−0.11	**−0.44**
MC			**0.71**	**0.80**	−0.08	0.29
SSP				**0.90**	**−0.59**	−0.23
Tot‐SH					**−0.40**	**−0.07**
Av‐SH						**0.80**
B: Smoking (*p* = 0.38)						
ST	**0.78**	**−0.97**	**−0.61**	**−0.76**	**0.66**	**0.53**
Temp		**−0.83**	**−0.82**	**−0.89**	**0.46**	**0.78**
MC			**0.67**	**0.78**	**−0.54**	**−0.59**
SSP				**0.94**	−0.23	**−0.78**
Tot‐SH					**−0.46**	**−0.78**
Av‐SH						0.32

Notes

Bold value denotes strong correlation.

Av‐SH, available sulfhydryl content; DT, drying time; MC, content; SSP, salt‐soluble proteins; ST, smoking time; Temp, temperature; Tot‐SH, total sulfhydryl content.

* Statistically significance.

### 3.3 Sulfhydryl groups and disulfide bond contents

**Figure 2:**
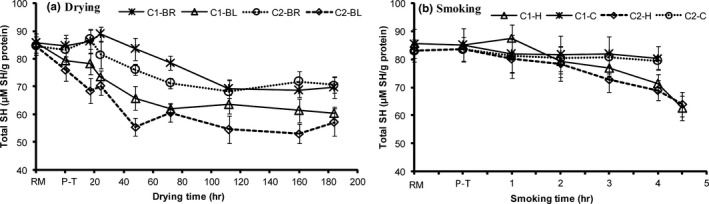
Changes in total sulfhydryl content in high lipid (C1) and low lipid (C2) capelin during drying and smoking (*n* = 3, 18 pieces [6 pooled together, in 3 bags]; Mean ± *SD*). Extraction of the protein fraction was performed in 1 M salt solutions. BL, blanched; BR, brined; C, cold smoking; H, hot smoking; P‐T, pretreated; RM, raw material

Generally, the total sulfhydryl (SH) groups content of the soluble muscle proteins significantly (*p* < 0.05) decreased after blanching, but a slight decrease was obtained in the samples after brining process (Figure 2a). During drying and hot smoking processes, the total SH groups of all samples significantly decreased (*p* < 0.05), except under cold smoking process where it remained relatively stable (Figure 2a,b). The total SH groups content of brined capelin remained remarkably higher than those of blanched capelin throughout the drying process (Figure 2a). In addition, lipid content had an influence on the total SH groups content in the samples during drying and hot smoking processes. A significantly higher total SH groups content was recorded in the C1 capelin when compared to those of C2 capelin (C1‐brined vs. C2‐brined, C1‐blanched vs. C2‐blanched and C1‐hot smoked vs. C2‐hot smoked). The decrease in total SH group content after blanching of capelin is understood to be due to the heat‐induced proteins denaturation. The salt diffusion into fish muscle during blanching may have had an effect on protein denaturation. The outcomes were in accordance with a decline in SSP of blanched capelin (Figure 1a). The decline in total SH groups content noted in capelin under both processing methods could have resulted from their vulnerability to oxidation instigated by of SH groups exposure from the native proteins during denaturation (Nguyen et al., 2011; Rawdkuen, Jongjareonrak, Phatcharat, & Benjakul, 2010; Zuazaga, Steinacker, & del Castillo, 1984), albeit concealing of SH groups by intrinsic particles/aggregates due to dryness may have also contributed.

**Figure 3:**
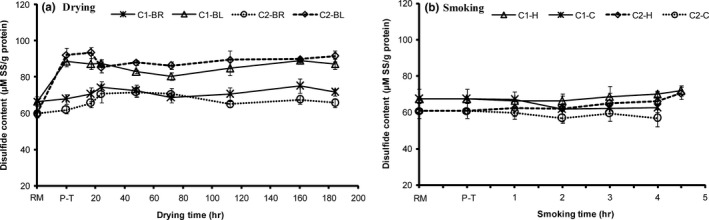
Changes in disulfide content in high lipid (C1) and low lipid (C2) capelin during drying and smoking (*n* = 3, 18 pieces [6 pooled together, in 3 bags]; Mean ± *SD*). Extraction of the protein fraction was performed in 1 M salt solutions. BL, blanched; BR, brined; C, cold smoking; H, hot smoking; P‐T, pretreated; RM, raw material

Brined C1 fish recorded the highest total SH content than the other sample groups during drying. Whereas blanched C2 fish had the least total SH content. Contrarily to the disulfide bond content observed in the groups, higher total SH content was obtained in brined C1 samples when compared to blanched C2 samples (Figure 3a). The lipids offered protection against proteins conformation decreasing the exposure of buried sulfhydryl groups to reactive oxygen and the by‐products of lipid oxidation. In contrast, blanching exposed the sulfhydryl groups in native proteins to the reactive oxygen or by‐products of oxidation. Blanching, therefore, enhanced sulfhydryl groups oxidation with the disulfide interchanges (formation of hydrogen and hydrophobic bonds) (Baylan et al., 2015; Hsu, Hwang, Yu, & Jao, 2007; Raman & Mathew, 2014). The slight increase in total SH content recorded in brined fish may have been instigated by modification of chemical groups, particularly SH, because of the initial alteration in protein–water interactions. Unlike the drying stages where surface water evaporated, water diffusion from the fish interior to the surface dominated in the later drying stages (Cyprian et al., 2015) leading to muscle proteins dehydration and aggregation.

**Figure 4:**
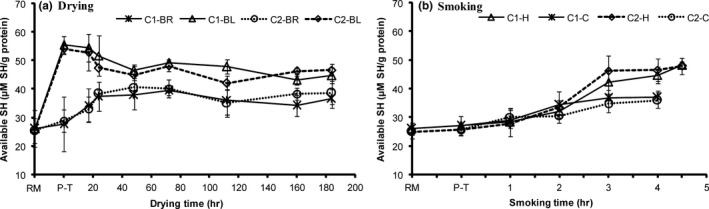
Changes in available sulfhydryl content in high lipid (C1) and low lipid (C2) capelin during drying and smoking (*n* = 3, 18 pieces [6 pooled together, in 3 bags]; Mean ± *SD*). Extraction of the protein fraction was performed in 1M salt solutions. BL, blanched; BR, brined; C, cold smoking; H, hot smoking; P‐T, pretreated; RM, raw material

The changes in sulfhydryl content recorded under hot smoking (Figure 4) could have been initiated by proteins aggregation other than oxidative reactions, as smoke is known to contain phenolic compounds which possess antioxidant properties (Guillen & Errecalde, 2002). Capelin displayed reduced content of lipid oxidation indicators after smoking (Cyprian et al., 2016). The concealing of sulfhydryl groups by proteins intrinsic particles or aggregates is thus suggested to have reduced the total sulfhydryl groups’ content during hot smoking, as it was relatively stable during cold smoking. During drying and smoking, a strong positive correlation was obtained between SH and moisture content, but also with temperature only during smoking (Table 2). This backs the elucidation of concealing sulfhydryl groups by inherent protein aggregates. Fish similarly pretreated (brined/blanched in brine) before drying or smoking obtained comparable total SH groups’ content, implying lipid had no influence on SH groups’ changes.

The available SH content increased after blanching and during early drying and smoking of brined fish (Figure 4a,b). It, however, increased most during blanching and at the end of hot smoking when temperatures were high. The available SH content depicted a tendency of declining at drying inception in blanched fish that had higher content than brined, correlating inversely with total SH groups content (Table 2, *r* = −0.40). The higher available SH content in blanched fish can be explained as earlier discussed by protein denaturation as blanching uncovered the buried sulfhydryl groups thus raising the fraction of available SH in the total SH groups contents. The observed increase in available SH content in brined samples at the initial drying stages could be linked to the salting‐in effect that might have led to swelling of proteins exposing SH groups (Baylan et al., 2015; Nguyen et al., 2011). According to Raman and Mathew (2014), the increase in SH in group may also be deduced to the reactive groups changes, predominantly hydrophilic surface loss and hydrophobic areas exposure. During early drying, available SH content reduced in blanched fish, steadying in all groups near the end of drying process. The masking of sulfhydryl groups by protein intrinsic particles/aggregates that formed due to tight proteins network caused by dryness is believed to have decreased the available SH content. The variations in available SH and disulfide bond contents were in conformity depicting a strong correlation (*r* = 0.80; Table 2).

Hot smoking increased the available SH content significantly (*p* < 0.05) when the temperatures were above 40°C. As explained earlier, high temperature during smoking led to protein denaturation, uncovering SH groups concealed in proteins. Nonetheless, owing to the smoke's antioxidant properties (Guillen & Errecalde, 2002), there were minimal disulfide interchanges in reactive SH groups portrayed by changes disulfide content that were not different (*p* > 0.05; Figure 3b & Table 2, *r* = 0.32). In general, available SH content merely correlated strongly with disulfide bond content during drying, but inversely correlated with moisture content (Table 2). Blanched capelin obtained significantly higher (*p* < 0.05) disulfide bond content, after pretreatment and during drying than in the other groups (Figure 3a). Brining appeared to have had minor influence on disulfide interchanges since a slight rise in content was obtained after brining and during initial drying. Afterward, disulfide content remained apparently stable in the groups. The results are in conformity those of total SH content in that blanched fish that obtained higher disulfide content had lower total SH content and vice versa for the brined samples. Sulfhydryl groups when exposed to reactive oxygen or secondary oxidative by‐products get oxidized consequently undergoing disulfide interchanges (Baylan et al., 2015; Hsu et al., 2007; Raman & Mathew, 2014), resulting in reduced total SH content and increased disulphide content. This suggests blanching aggregates proteins thereby increasing the disulfide bonds while reducing total SH groups’ content. At latter drying stages, disulfide bond content was steady corresponding with the stability in SH groups’ content. Lipid content did not affect the available SH and disulfide content significantly during processing (drying and smoking) albeit C1 capelin depicting a tendency of higher SH content and lower available SH content than C2 capelin.

## 4 CONCLUSIONS

The pretreatments (brining and blanching in brine), processing (drying and smoking) methods, and the lipid content were found to influence proteins conformational changes in capelin. The SSP and total SH contents reduced, whereas the available SH and disulfide bond content increased during the experimented fish processing. Protein conformational changes were reported to have occurred during fish drying, predominantly when moisture content and dehydration rate were comparatively high. Capelin blanching pretreatment prior to drying decreased protein solubility and SH content due to protein aggregation which may have affected the sensory properties of the dried fish mainly texture. During smoking, conformational changes occurred mainly under hot smoking. The changes were ascribed to protein denaturation that reduced fish muscle water holding capacity affecting the yield, which is of a key interest to fish producers. As was expected, cold smoked samples recorded fewer changes in protein structure.

Less changes in protein structure (SSP and SH groups) were obtained in C1 (fatty) capelin during both experimented processing methods, implying lipids have a protective effect on capelin proteins. Protein conformational changes were greater in blanched and during drying than in brined and changes during smoking. Capelin is therefore not recommended for blanching pretreatment in commercial drying. Nonetheless, drying of fish ought to be conducted under low temperature conditions to maintain nutritive value and sensory qualities.

## CONFLICT OF INTEREST

The authors have no conflict of interest to declare.

## ETHICAL APPROVAL

In the work herein, we confirm that no human subjects were involved and thus human and animal testing was unnecessary.
